# Absence of menstruation in female athletes: why they do not seek help

**DOI:** 10.1186/s13102-021-00372-3

**Published:** 2021-11-23

**Authors:** Saskia J. Verhoef, Merel C. Wielink, Edwin A. Achterberg, Marlies Y. Bongers, Simone M. T. A. Goossens

**Affiliations:** 1 Faculty of Health, Medicine and Life Sciences, Maastricht University, Universiteitssingel 40, 6229 ER Maastricht, The Netherlands; 2 Department of Obstetrics and Gynaecology, Máxima Medical Centre, PO Box 7777, 5500 MB Veldhoven, The Netherlands; 3 Department of Sports Medicine, Máxima Medical Centre, De Run 4600, 5504 DB Veldhoven, The Netherlands; 4 Research School Grow, Maastricht University, Universiteitssingel 40, 6229 ER Maastricht, The Netherlands; 5 Eindhoven MedTech Innovation Center (E/MTIC), Horsten 1, 5612 AX Eindhoven, The Netherlands

**Keywords:** Amenorrhea, Relative Energy Deficiency in Sport (RED-S), Athletes, Sports

## Abstract

**Background:**

It is known that amenorrhea is highly prevalent among female athletes. However, a large percentage of them do not seek help if this complaint occurs. We performed this study to gain more insight into the reasons why female athletes do not seek help when experiencing amenorrhea and how care for these women could be improved.

**Method:**

Qualitative focus group research. Female athletes were approached to take part in a focus group. They were asked about the main reasons for not reporting amenorrhea and how care for amenorrhea, in their opinion, would ideally be organised. The women were asked to make a list of their top five reasons for both subjects and discuss this among their peers.

**Results:**

According to the participants, the five main reasons for not reporting the amenorrhea were: (1) normalizing of the subject, (2) the absence of menstruation is not perceived as a problem by the athletes themselves, (3) experienced shame and taboo, (4) prioritisation of sports performance, and (5) denial. Factors to improve care around menstrual cycle problems in female athletes were: (1) informing athletes, coaches, trainers and mentors, (2) informing doctors, (3) conducting more research on long-term consequences, (4) breaking the taboo on menstrual problems, and (5) having a multidisciplinary collaboration between different specialisms.

**Conclusion:**

By informing athletes, coaches, trainers, and mentors about menstrual cycle problems in athletes, more awareness among those groups can be created. According to the athletes, more research is needed on the long-term consequences of amenorrhea in sports, to enable them to make a better assessment of their possible future health risks. Women experience a taboo on discussing menstrual problems; role models discussing the problem may help in decreasing the taboo. A multidisciplinary collaboration of health care providers may improve care around female athletes with menstrual problems.

## Background

More and more women are participating in sports, both at recreational level and professional level. The Olympic Games in Tokyo were the first Olympic Games in which almost 50% of the participating athletes were women [1]. For the last year, there has been an increasing focus on women in sports, including in scientific literature. One of the topics receiving more attention is RED-S (Relative Energy Deficiency in Sport). RED-S was introduced in 2014 and replaces the term ‘Female Athlete Triad’. One of the reasons to replace the Female Athlete Triad with RED-S, is that RED-S not only involves female athletes, but also male athletes. However, since menstrual problems, both amenorrhea and oligomenorrhea, are one of the most well-known consequences, the syndrome might be easier to diagnose with female athletes than with male athletes [2–4].

The etiological factor of RED-S is a low energy availability (LEA), caused by an imbalance between energy intake and energy consumption. A LEA can be the result of disordered eating or eating disorders, but can also occur without disordered eating or eating disorder. The energy imbalance results in a disruption of various physiological functions and systems such as the basal metabolism, menstrual cycle, the (build-up of) bone density, immune system, and cardiovascular health. The most evident symptoms that can occur are menstrual cycle abnormalities such as amenorrhea or oligomenorrhea. Other symptoms include stress fractures, but also constipation, cardiac arrhythmias, and reduced muscle strength. The exact prevalence of RED-S is unknown. One of the long-term risks is a decreased mineral bone density. Other potential long-term risks are also still largely unknown [5–10]. Psychological problems, such as anxiety, irritability and depression play an important part as they can both lead to RED-S and be the result of RED-S. Also, there can be an overlap between eating disorders and LEA; eating disorders can lead to LEA and LEA can also result in eating disorders [9–11].

Langbein et al. recently demonstrated the complexity of psychological and physiological interactions of the development of RED-S in a qualitative study among endurance athletes. They also suggested that psychological factors may exacerbate the syndrome or delay recovery [11].

It is known that female athletes underreport menstrual cycle disorders, with a prevalence of up to 40% in some studies [4]. Miller et al. found that 22% of athletes in lean-build sports would not report amenorrhea [12]. In a recent research questionnaire, among female athletes in the fertile life stage in the Netherlands by our study group, 15.6% of all (semi)professional athletes (on average 15 h of training per week) who did not use hormonal contraception indicated that they did not have a menstrual period for the last six months. Of all responders, 31% indicated they would not seek help if they developed unexplained amenorrhea (unpublished results). Several studies have also reported on low awareness among female athletes and their trainer or coaches of the potential healthcare risks that are related to the RED-S syndrome [5, 6, 13].

This focus group study aimed to gain insight into reasons why female athletes do not seek help with the most evident symptom of LEA; amenorrhea, and what the ideal care for this problem would look like for them. A focus group study design was chosen to explore a wide range of views and experiences on the topic [14, 15]. These insights can help to reach this group of female athletes, coaches and sports federations, better in the future and improve the care around menstrual problems in athletes.

## Method

For this focus group interview study, we asked women who had previously indicated that they were interested to participate in a study on the topic ‘menstruation and sports’. These women had filled out a research questionnaire, focusing on menstruation (disturbances) and sports performance. For this questionnaire, every national sport association recognized by the Dutch Olympic Committee was asked to distribute the questionnaire among their members through their social media accounts, periodical e-mails and websites. In addition, the questionnaire was brought to the attention through social media (Facebook, Twitter, LinkedIn and Instagram). Some (ex-) professional athletes and social influencers were asked to share the questionnaire on their social media accounts as well. The questionnaire was online from January-March 2020.

Women who answered to be interested to participate in further research, were considered potential participants for our focus group study. Potential participants were asked by email to participate in a focus group. Participants were selected based on sports background to provide a diverse range of (endurance) sports backgrounds and ages.

In preparation for the focus groups, an interview guide was developed by two of the researchers (MW and SG). First a brainstorm was held to decide on the two main topics (reasons not to seek help and factors to improve healthcare) and on possible open questions. Next, questions were deleted or rephrased until both researches decided the interview guide was completed [16]. We did not perform a pilot focus group.

All participants gave both verbal and written informed consent and agreed to the recording of the focus groups. The Daily Board of the Medical Ethics Committee of Máxima Medical Centre confirmed that the rules laid down in the Medical Research Involving Human Subjects Act do not apply to this research (METC-number N20.067).

The semi-structured focus groups were led by two of the researchers (MW and SG) based on the pre-developed interview guide (Table 1). MW and SG were instructed by a colleague with extended experience on facilitating focus groups.

**Table 1:** Interview guide

Structure of focus group
*1. Introduction*
Introduction of facilitator and researchers and their occupation and background, personal interest in sports and explain study goal and basic rules
Invitation to the participants to introduce themselves
What is your main reason for participating in this focus group?
*2. Make an inventory of reasons why women do not seek help if they do not menstruate*
Why do women not seek help when they do not menstruate?
Are there any reasons missing from this list [show list of reasons stated by the participants]?
What are the five most important reasons according to you?
*3. Make an inventory of factors to improve the care about menstrual problems of athletes*
How do you think the care around menstrual problems of athletes could be optimized?
Are there any reasons missing from this list [show list of reasons stated by the participants]?
What are the five most important reasons according to you?
*4. Closing and word of thanks*
Additional questions to support the discussion
Why is this an important feature?
What is the effect of this feature?
Do other participants recognize this point?

**Fig. 1:**
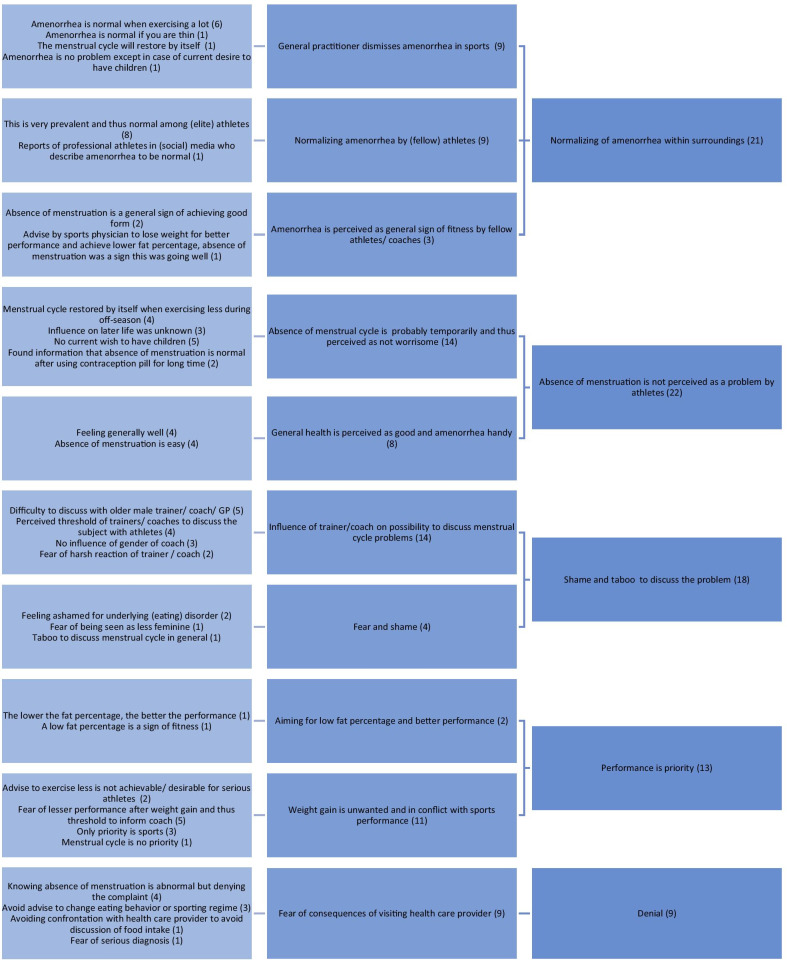
Coding tree reasons for not reporting the absence of menstruation

**Fig. 2:**
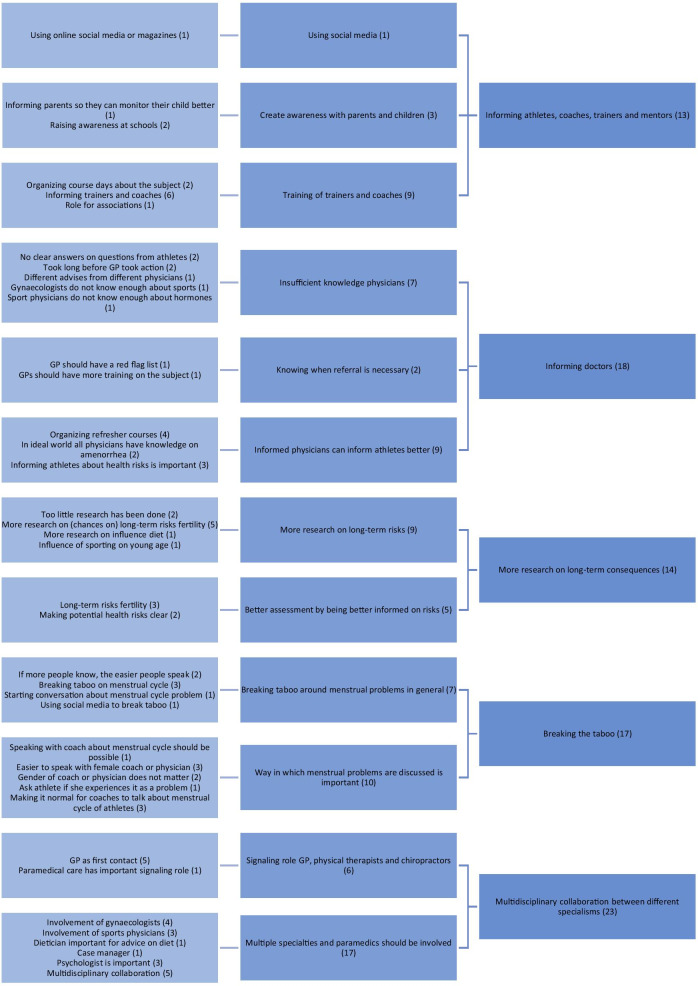
Coding tree optimization of care around menstruation problems in female athletes

The groups met online via the web videoconferencing platform ZOOM due to the COVID-19 measures. Participants joined using both video and audio. Participants were not reimbursed for their participation. All researchers and facilitators participating in the focus group were female, to exclude the possibility of participants to feel a threshold to discuss their menstrual cycle due to the presence of a male researcher. No other people besides the participants and researchers were present. In the introduction, the researches introduced themselves briefly, including their name and occupation (SG: gynaecologist, MW: sports physician in training, SV: medical student), their personal sports background and reason to initiate or participate in the research. Furthermore, the goal of the research was introduced. Two researchers (MW and SV) took notes during the focus groups (Tables 2, 3). The focus groups took place in May and June of 2020 and lasted approximately 90 min. The focus groups were audio recorded digitally and transcribed by one of the researchers (SV). The recordings were deleted after transcription was completed. The transcripts were coded using Microsoft Word and Microsoft Excel. The items (top five) of the sub questions were derived from the data and divided into different categories and subcategories by three researchers (SV, MW and SG) based on thematic analysis. Two coding trees were developed (Figs. 1, 2). The focus groups were done one by one until all authors agreed saturation of input was reached. No further repeat interviews were carried out. The COREQ checklist was used to ensure thorough reporting [17].

**Table 2:** Reasons that were mentioned not to report absence of menstruation (in bold; most important factors according to the corresponding group)

Group 1	Group 2	Group 3
**Is not seen as a problem**	Fear of something serious	**Do not worry**
**It is not known what consequences will be later in life**	‘Denial’	Perceiving it as normal
**Will go away, general practitioner says ‘it is part of the game’**	**Thinking it is normal**	Experiencing no menstruation as convenient
Little attention from mentors for this problem (in certain sports/certain federations)	**Shame and taboo**	Showing you are fit
No direct supervision of team doctor (in certain sports/levels)	Little confidence in Western healthcare	**No desire to have children**
Sign of getting in shape	**Ignorance**	**Not easy to discuss with (male) trainers**
**Appears to be reversible, therefore less worries**		Not common for coaches to ask about it
Difference between top-level sports and recreational sports: detect and report subtle signals		**Other priorities**
**Shame**		More common among teammates
		**Not wanting to hear it**/**denial**
		Seeking help late by athletes in general

**Table 3:** Factors that were mentioned to optimize care around menstrual problems in athletes (in bold; most important factors according to the corresponding group)

Group 1	Group 2	Group 3
**Informing federations/coaches/trainers/mentors**	**Educate general practitioners**	**Informing coaches/trainers, responsibility**
Fixed team doctor/trainer etc	Care for non-top athletes	**Realistic image of consequences**
**Gynaecologist with sufficient knowledge early in the process**	Create awareness/break taboos through social media/presentations	Starting the conversation with athletes
Sports doctor with the right knowledge	**Informing coaches through sports federations**	Confidential adviser
**Mentioning concerns about the future/fertility**	No clear difference between male and female doctors	**Education/informing**
Female doctors/care providers	Helicopter view	Discuss risks with athlete/educate
Red flag list at general practitioner, refer to specialist when necessary	Attention to nutrition/eating problems	Involve sports physician
**Shared clinic/consultation: gynaecologist + sports physician**	**Informing parents**	Involve psychologist
Other specialisms: gastroenterologist, endocrinologist, pulmonologist	**Breaking the taboo/being taken seriously**	Involve sport dietician
**Casemanager**	**Knowledge in schools**	Signalling function for trainer and paramedic care
		Regionally bound network
		**Breaking the taboo; must become normal to discuss it**
		Role model who does menstruate
		Better background information/guidelines for general practitioner

The main goals of the focus group were to (1) identify reasons why female athletes do not seek help in case of unexplained amenorrhea and (2) to identify factors to improve (health)care for female athletes suffering from these complaints.

## Results

A total number of 16 women were asked to participate on the study group. Twelve participants agreed to enter the focus group. The mean age of the participants was 33 years (range 23—45). The participants (previously) practised mainly cycling (n = 6), triathlon (n = 4), speed skating (n = 4), athletics (n = 2), running (n = 2) and mountain biking (n = 1). A number of women (n = 6) practiced multiple sports. Half of the participants (n = 6) were (ex) elite or professional athletes.

A total of three focus groups took place, each with four Dutch female participants. After the third focus group, the researchers decided no new information was provided, appropriate to data saturation.

### Reasons for not reporting the absence of menstruation

The five most important reasons for not reporting the absence of menstruation derived from the interviews were (1) normalizing of amenorrhea within surroundings, (2) the absence of menstruation is not perceived as a problem by athletes, (3) shame and taboo, (4) prioritising performance and (5) denial. Each reason is further described below, supplemented by indicative quotes. The quotes were translated from Dutch, with F = focus group number and P = participant.

#### Normalizing within surroundings

Participants indicated that one of the main reasons for not reporting amenorrhea was normalization by doctors, coaches and other athletes. Some participants mentioned that the general practitioner told them in the past that the absence of menstruation was ‘part of exercising a lot’, so these women did not report this complaint to the general practitioner afterwards (F2, P2: *“Especially, if you have reported it [the absence of menstruation] and a doctor says that it is okay, then you will certainly never raise the alarm again, even if you do not get it for much longer.”*). In addition, these women knew several (elite) athletes who did not menstruate, so they thought this was a normal phenomenon, or a sign of fitness (F3, P1: *"Because it's very much a shared thing [absence of menstruation], so it is, yes, nobody really worries about it. It's kind of seen as normal anyway, I think, if you are very fit then that is something that comes with it." F3, P3: “In the world of athletics, the girls I trained with at the time, it was also a bit of a thing sometimes, to say, to show that you were fit. Because then you also had a low- fat percentage. It was actually used more among the girls themselves, so to speak, as ‘look I am super fit, because I do not even have my periods anymore’.”*).

#### The absence of menstruation is not perceived as a problem by the athletes themselves

Another reason was that many participants did not experience the absence of menstruation as a problem. For example, women thought it was convenient and also because they had no current whish for children. (F3, P2: *"It is the case with me and it [the menstruation] stopped at one point. And yes, I did not really see the seriousness of that. It was like that for a number of years. One, because I found it very easy and did not, did not immediately notice or feel anything about it. So I was not worried either, I did not have a desire to have children."*). In addition, they trusted that menstruation would return when decreasing exercise intensity (F1, P4: *“Because in the winter, when it was of course relatively quiet with competitions, then I noticed that maybe I was not having my period very regularly, but I was just getting my period. And that it was mainly during the summer period with a lot of competitions in which this was absent.”*). The participants indicated that there was an ignorance about the consequences of the absence of menstruation (F1, P4: *“You also do not know how it will affect you later in life, because that education is not available.”* F2, P2: *“Because the fact that I did not report it at the time was really because I genuinely did not know that it was so bad so to speak. And I think there are really a lot of people who do not know that either.”*).

#### Shame and taboo

For some participants, shame and taboo played a role. Women were ashamed of not having their period because they were afraid of being judged for a lack of femininity (F2, P1: *“That you are judged on that, that it does not happen, that it does not happen, well that something is wrong then because every woman basically gets her period every month and if it does not happen then maybe there is something wrong with your femininity.”*). Also, in some cases there was an underlying eating disorder, which was a difficult topic for them to discuss (F2, P1: *“That was partly due to sports and partly an underlying eating disorder I developed at the time” … “For me, it was mainly shame at the time why I did not raise the alarm. Because a lot of other things were also involved.”*).

Several participants experienced a taboo around discussing menstruation (F2, P4: *“And I certainly think that talking about it is a taboo. That a lot of women still feel like, I would rather not.”*). There were different opinions about the relevance of the gender of the care provider or coach regarding this topic (*F1, P2: “Now I have a female general practitioner and before I had a male general practitioner. I found it less pleasant to discuss something like that with a 60- year old man than with a woman.” F2, P2: “I have always had male coaches and I personally do not have a problem talking about that… So I feel like that is a much discussed topic. So whether that is a woman or a man does not really matter to me.”*).

#### Performance is priority

According to the participants, performance is an absolute priority in an elite athlete’s life. Therefore, as young adults, they only had an eye for sport (F3, P3: *“In an elite sports life or in a phase of that part so to speak, yes, then your goal is to perform… then that is number one. And it really is not anything else. Then you are not concerned with having children or anything else I think.”*). According to the participants, performance was also a priority for coaches at the highest level. Therefore, athletes experienced an additional barrier to approach a coach or doctor with any health concerns (F1, P4: *“I have to live with it because then I perform better. And if I tell my coach that I have this problem which means I actually have to weigh more, then I will not perform the way I normally would. So of course that creates a huge threshold to present something like that to a trainer or coach.”*).

#### Denial

Some participants knew that a lack of menstruation was not a good sign but did not want to be confronted with this. The elite athletes were afraid that they would have to make adjustments in their diet or training regimen that might worsen their performance (F3, P1: *“That you are afraid that if you go to a doctor, that he or she will tell you to change something. So getting fatter, gaining weight, becoming less fit. So that if you go to get help, that someone is actually going to tell you what you do not want to hear.” F3, P2: “It is kind of that it is not normal, but especially something would have to change in your pattern… Especially the fear of that. That exactly… No I would not have wanted to hear that, for example, at the time when I was really sharp, I think at that time I would have especially not wanted to hear that I should have gained, say, 5 kilos.”*).

### Optimization of care around menstruation problems in female athletes

Participants mentioned several factors that could contribute to an optimization of care around menstrual problems in athletes. The five most important factors distilled from the interviews were (1) informing athletes, coaches, trainers, and mentors, (2) informing general practitioners and other health care providers, (3) conducting more research on long-term consequences, (4) breaking the taboo on menstrual problems, and (5) developing multidisciplinary collaboration between different health care specialists.

The factors are explained in detail below.

#### Informing athletes, coaches, trainers and mentors

According to the participants, athletes, coaches, trainers and mentors should all be informed about the significance of absence of menstruation in female athletes so that awareness can be created. For example, athletes could be reached through social media or be educated at schools (especially schools for elite athletes).

Informing coaches or trainers during for example course days was considered important because of their signalling function (F1, P4: *“Informing coaches, trainers and guidance about this. Those [coaches, trainers] are the people who have to signal it, or at least with whom a cyclist has to feel safe enough to discuss that problem. And that is only possible if cyclists know that this problem has also been mentioned during courses.”*). Participants indicated that athletes often feel safe with their coach or trainer, and therefore they should (be able to) start a conversation about menstrual problems. Informing the parents of athletes was also mentioned, because they can monitor the development of their child (F2, P1: *“If they [parents] also know more about it, suppose, perhaps, being informed by coaches who indeed know more about it, then they can also keep more of an eye on it. Because they of course see their child every day, so to speak, and they might also talk with them more.”*) and identify changes.

#### Informing doctors

According to the participants, there is still a lot of progress to be made in informing doctors (F1, P1: *“I notice that I still have to find out a lot of things myself and then I present it to both my gynaecologist and sports physician, then they will have a look at it. But that there is still a lot of ignorance.”*

*F2, P1: “At the time [of amenorrhea] I was advised by one *general* practitioner to just take the contraception pill to induce it, and the other general practitioner said ‘you actually should not do that, your body should do that on its own.’”*), for example, by organizing refresher courses or (additional) trainings about RED-S. Participants expected this would enable physicians to better inform athletes. Some participants indicated that a general practitioner or physician does not need to know all the details about the subject but does need to know when a referral is necessary (F1, P1: *"But maybe a general practitioner can have some kind of a red flag list, for when should I refer in such cases."*).

#### More research on long-term consequences

Participants indicated that they would like to be educated on the long-term consequences, especially on fertility (F2, P2: *"Because I think I am most afraid that my elite sport would do something to my fertility, yes. Because that is really, yes I would find that very bad, so to speak."*). They indicated that more research should be done on this subject (F3, P3: *"When someone comes, what can you tell them? What are the rates of infertility, I am just saying something, how likely is it that the menstrual cycle will just come back?"*). According to participants, this would allow an athlete to be better informed and to make a better assessment of the risks (F3, P3: *"The ultimate goal is maybe to provide insight into what it can cause or what it can mean for the future, to provide insight into the dangers."*).

#### Breaking the taboo

Participants repeatedly mentioned the importance of breaking the taboo surrounding menstrual problems (in athletes). In part, this could be achieved by making the existence of the problem known to a wider audience (F2, P4: *"And the more people know about this, that this is an issue at all, the more people will also be interested in it and will talk about it more easily."*). The way in which menstrual problems are discussed with an athlete by a coach or doctor is important according to the participants (F3, P3: *"I think the way you start the conversation matters a lot. For example, you can say 'I know of women with a lot of injuries that they very often do not have their periods, that actually happens quite often, is that the same for you?' That is already a different question than simply asking an open question, I think."*) to find out how an athlete can be helped in the best way (F3, P3: *"That is actually the most important thing, that you start the conversation. What belief does someone have, what idea does someone have about it? I think that is the most important thing."*).

#### Multidisciplinary collaboration between different specialisms

According to the participants, a general practitioner ideally plays an important role in the care surrounding amenorrhea. Besides a signalling role, the general practitioner could assess what kind of help an athlete exactly would need (F3, P3: *"I think that is just the job of the general practitioner so to speak. That you are going to look at what is the request for help and what is the problem, so to speak, and who can I involve."*). Physical therapists and chiropractors may also have a signalling function (F3, P1: *“I think many athletes also see physiotherapists, chiropractors, a bit of paramedical care. I could imagine that they also have a signalling function. They see you, quite simply, in your underwear so they can see how you are, how trained you are or not. Certainly at the moment when you are lying there on the couch every week, then they might be someone who can start that conversation much easier than, for example, a general practitioner who you see once in a while."*). Participants additionally mentioned the importance of a multidisciplinary collaboration between different specialties and paramedics, including a gynaecologist, sports physician, sports dietician, and psychologist (F1, P4: *"That a gynaecologist and sports physician communicate about this together about the effects of sports on hormones."* F3, P1: *"And then there is a small group that might want to do things differently, but it does not work out, I would perhaps rather refer them to a psychologist than to a gynaecologist. Because I think that if it is really about being well-trained, being light, being good, yes, in my opinion there is much more of a psychological aspect to it than a gynaecological one."*).

## Discussion

### Reasons for not reporting the absence of menstruation

According to the participants, the main reasons that female athletes with amenorrhea do not seek help were 1) normalization of amenorrhea, 2) not experiencing the absence of a menstrual cycle as a problem, 3) experienced shame and taboo around the subject, 4) prioritisation of sports performance, and 5) denial of the problem.

Both normalizing amenorrhea and not experiencing amenorrhea as a problem are vicious circles that can be broken by educating female athletes and those around them about RED-S and the associated potential health problems [6].

Participants indicated that they experience a taboo on menstrual problems. By breaking this taboo, there will be less embarrassment for athletic women to seek help for amenorrhea. Social media influencers and role models could play an important role to achieve this. In recent years, big players like Garmin and Nike have started to motivate women to track their menstrual cycle in their mobile app. This might also lead to more awareness among female athletes.

Failure to ask for help when not menstruating because performing is a priority, is often an incorrect assumption. RED-S also has negative effects on athletic performance, which can include decreased muscle strength, decreased endurance, and increased risk of injury [6]. Training sessions are also more likely to be cancelled due to injuries, illnesses, or complaints, resulting in a reduced training effort. Also the psychological impairments associated with RED-S will negatively influence sports performance. Thus, by better informing athletes about RED-S, incorrect assumptions and fears can be removed.

### Optimization of care around menstruation problems in female athletes

The main factors that participants mentioned for optimizing care around menstruation problems in female athletes were informing athletes, coaches, trainers, and mentors, informing general practitioners and other health care providers, conducting more research on long-term consequences, breaking the taboo, and developing multidisciplinary collaboration between different specialisms.

Little attention is paid to RED-S within international sports federations. In 2017, only 7% of international sports federations had a program or guideline on RED-S or conducted research in this area [18]. The IOC recommended improving awareness of RED-S among athletes, coaches, mentors, and sports organizations [6].

Recently, Langbein et al. have described a qualitative exploration of RED-S in endurance athletes. While their patient population differed somewhat of our population and the study focused on different aspects, they also report on the lack of information received and the need for professional support by the athletes, and emphasize on the importance of well-informed members of the team (parents, coaches, trainers, physicians) surrounding the athlete [11].

As far as is known, no research has been conducted into long-term consequences and fertility. The IOC cited the long-term consequences of RED-S as one of the major gaps in knowledge about this syndrome [6].

For several athletes, a multidisciplinary collaboration between different specialties, including a gynaecologist, sports physician, sports psychologist, and sports dietitian, would be ideal for the care of RED-S. A signalling role is mainly reserved for coaches, trainers, physiotherapists, and general practitioners. Appointing a multidisciplinary team to treat RED-S is also a recommendation given by the IOC [5].

### Strengths and weaknesses

This focus group study provides insight into the reasons that female athletes do not report amenorrhea and how care can be improved around menstrual cycle problems. These insights were provided by women of different ages and different athletic backgrounds. The heterogeneity of the participants results in a better generalisability of the results.

On the downside, some women shared experiences from the past. It is possible that the taboo on speaking about menstrual cycle disorders they experienced in the past has already decreased somewhat. Also, recall bias might have occurred. Moreover, it is likely that knowledge and experience of doctors and coaches about menstrual problems in athletes have increased in recent years.

Amenorrhea is not the only possible form of menstrual dysfunction in RED-S, also other menstrual cycle disorders such as oligomenorrhea may occur. Furthermore, a range of different symptoms are associated with RED-S. These symptoms were not investigated in this focus group study.

Due to the nature of the recruitment of participants, it is possible we have created a selection bias. Also, we focused mainly on endurance athletes, while RED-S and menstrual cycle problems may occur on different types of sports as well.

Transcripts were not sent to participants for comment and/ or correction, nor were the participants able to provide feedback on the findings. The final manuscript was sent to the participants.

## Conclusion

The reasons that were given by the participants for not reporting the absence of menstruation are (1) normalization within the environment, (2) not perceiving the absence of menstruation as a problem by the athlete, (3) perceived shame and taboo on the subject, (4) prioritisation of sports performance, and (5) denial.

Several options to improve care around menstrual cycle problems in athletes emerged from this study. More awareness may be created by informing athletes, coaches, trainers, mentors, and sports associations. General practitioners and other health care providers should receive additional training to better inform athletes. In addition, more research on long-term consequences (especially fertility) will help athletes to make a better assessment of the risks in the future. Finally, the care of RED-S can be improved through a multidisciplinary collaboration between different specialties and paramedics. The Máxima Medical Centre has now started a monthly multidisciplinary consultation with sports physicians, sports dietitians, and gynaecologists on women with these complaints.

## Data Availability

Data are presented in the manuscript and in the attachments. The transcripts of the focus groups are stored on a protected disk in Máxima Medical Centre.

## Abbreviations

RED-S: Relative energy deficiency in sport
LEA: Low energy availability
